# Au Micro‐ and Nanoelectrodes as Local Voltammetric pH Sensors During Oxygen Evolution at Electrocatalyst‐Modified Electrodes

**DOI:** 10.1002/smsc.202300283

**Published:** 2024-02-12

**Authors:** Lejing Li, Ndrina Limani, Rajini P Antony, Stefan Dieckhöfer, Carla Santana Santos, Wolfgang Schuhmann

**Affiliations:** ^1^ Analytical Chemistry – Center for Electrochemical Sciences (CES) Faculty of Chemistry and Biochemistry Ruhr University Bochum Universitätstr.150 D‐44780 Bochum Germany

**Keywords:** Au micro/nanoelectrodes, electrocatalysis, local pH, oxygen evolution reaction, scanning electrochemical microscopy

## Abstract

The scarcity of state‐of‐the‐art oxygen evolution reaction (OER) electrocatalysts has led to intensive research on alternative viable electrocatalytic materials. While activity and cost are the main factors to be sought after, the catalyst stability under harsh acidic conditions is equally crucial. Considering that OER is a proton‐coupled electron‐transfer reaction that involves local acidification of the reaction environment by liberation of H^+^, the catalyst stability can be largely compromised in such conditions. Consequently, probing the pH value near the catalyst surface under operation leads to a deeper understanding of this process. The applicability of bare Au microelectrodes and nanoelectrodes as sensitive local pH probes during OER is shown in this work by using scanning electrochemical microscopy (SECM). Two case studies are presented, including the state‐of‐the‐art OER catalyst (IrO_2_) in acidic media and a ZnGa_2_O_4_ catalyst in alkaline buffered solution, demonstrating the suitability of the Au probe to accurately determine the local pH value in a wide pH range.

## Graphical abstract

Au micro‐ and nanoelectrodes were developed as local voltammetric pH sensors during the oxygen evolution reaction (OER). Case studies including IrO_2_ in acidic media and ZnGa_2_O_4_ in alkaline buffer solution demonstrate the suitability of the Au tip to accurately determine the local pH value in a wide pH range and buffering environments.

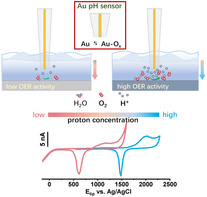

## 1 Introduction

Water splitting is undoubtedly one of the most recalled and auspicious topics in energy conversion and storage technologies, comprising of hydrogen evolution reaction (HER) and oxygen evolution reaction (OER). The latter is one of the impediments in this field mainly due to its complex mechanism that depends on the current density and overpotential.^[^ ^1^ ^]^ The high overpotentials required to liberate molecular O_2_ (Equation (1)) require highly stable, active, efficient, and pH‐universal OER electrocatalysts.^[^ ^2^ ^]^ The intrinsic acidification during OER in acidic (Equation (1)) and alkaline (Equation (2)) conditions makes most OER catalysts liable to such changes in pH, compromising their stability and complicating the interpretation and comprehension of the measured results. For example, modeling studies have previously shown that drastic local pH changes occur during the OER at neutral conditions due to a possible switch between the acid and alkaline mechanisms.^[^ ^3^ ^]^ This highlights the significance of determining the local acidity and basicity near the catalyst interface, so one can take adequate measures to tailor the catalyst material and have insights into the mechanistic nature of the reaction.^[^ ^4^ ^]^

(1) $$2\;H_{2}O⇌O_{2}+4\;H^{+}+4\;e^{-}\;\;\;\;\;\;\;\;\;\;\;\;1.23\text{ V vs. SHE}$$

(2) $$4\;{\text{OH}}^{-}⇌O_{2}+2\;H_{2}O+4\;e^{-}\;\;\;\;\;0.40\;V\;\text{vs. SHE}$$

The local change in pH can be measured with macro‐scale electrochemical methods such as rotating‐ring‐disc‐voltammetry^[^ ^5^ ^]^ where the IrO_x_‐modified ring electrode can be used as a potentiometric pH sensor showing a Nernstian/super Nernstian open circuit potential (OCP) as a function of pH.^[^ ^6^ ^]^ The possible dissolution of IrO_x_ in highly acidic media may, however, be problematic.^[^ ^7^ ^]^ In addition, macroscopic potentiometric sensors often have a long response time, and their response can suffer from interferences when employed in an electric field (e.g., two working electrodes), making it more challenging to deconvolute the OCP used as a pH‐shift indicator.^[^ ^8^ ^]^ Scanning electrochemical microscopy (SECM) as a micro/nanoscale method, besides being used as a tool for OER investigations via the substrate generation/tip collection mode,^[^ ^9^ ^]^ contributed significantly to investigations concerning the local pH value. SECM has the unmatched advantage of higher resolution and sensitivity, enabling the measurements at closer proximity to the catalyst surface.^[^ ^10^ ^]^ SECM was utilized to probe the time‐dependent local pH changes during HER^[7a]^ and ORR,^[^ ^11^ ^]^ during the CO_2_ reduction reaction (CO_2_RR),^[^ ^12^ ^]^ and in studies elucidating the influence of the local pH on the CO_2_RR product selectivity.^[^ ^13^ ^]^ Pt probes have been widely used as voltammetric pH sensors in such measurements due to the high sensitivity of the dependence of the potential of the Pt/PtO redox conversion to the variation of H^+^ and H_2_O activities at the tip environment.^[^ ^14^ ^]^ However, knowing that Pt probes are also very good catalysts to reduce O_2_, they cannot be used during O_2_‐producing reactions such as the OER. An alternative choice of a probe material could be Au, whose oxidation process (described in Eq. 3 for the lower pH range)^[^ ^15^ ^]^ exhibits similar high sensitivity to H^+^ and H_2_O activity variations. Specifically, the increase in proton activity induced by the OER causes a positive shift of the Au/Au_2_O_3_ redox potential according to the corresponding Nernst equation (Equation (4)). Therefore, the potentials at which these redox conversion peaks appear in cyclic voltammograms (CV) can be used to elucidate the local pH near the operating catalyst surface. Functionalized Au probes were used previously in local pH studies, however, the stability of such redox probes in acidic media remains insufficient.^[7a]^

(3) $$2\;\text{Au}+3\;H_{2}O⇌{\text{Au}}_{2}O_{3}+6\;H^{+}+6{\text{ e}}^{-}\;\;\;\;1.46\;V\;\text{vs. RHE}$$

(4)

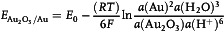




In this work, local changes in H^+^ and H_2_O activity were measured in close proximity to the operating OER catalyst surface by using a bare Au micro/nanoelectrode as a SECM tip which shows preserved stability even in highly acidic environments. The state‐of‐the‐art OER catalyst IrO_2_ as well as a poor OER catalyst (ZnGa_2_O_4_) were used as case studies to demonstrate pH variations at acidic and basic‐buffered conditions, respectively, demonstrating the wide range of applicability and high sensitivity of the suggested probe to local pH changes.

## 2 Results and Discussion

**Figure 1:**
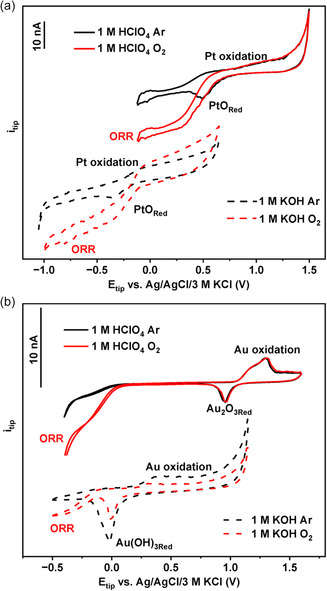
a) CVs of a Pt microelectrode (≈25 μm in diameter) in 1 m HClO_4_ and 1 m KOH. b) CVs of an Au microelectrode (≈10 μm in diameter) in 1 m HClO_4_ and 1 m KOH. All experiments were recorded at a scan rate of 200 mV s^−1^ in Ar and O_2_‐saturated solutions.

Considerable advances were made using bare Pt electrodes for local pH probing,[^13^, ^16^] based on the well‐known characteristics of polycrystalline Pt surfaces in acidic media.^[^ ^17^ ^]^ However, the determination of the shift of the PtO_Red_ reduction wave in the voltammogram is compromised if OER occurs at the sample electrode due to the high local oxygen partial pressure which leads to an overlapping voltammetric response due to the low‐overpotential oxygen reduction reaction (ORR) at the Pt probe^[^ ^18^ ^]^ as depicted in **Figure** **1**a. The convolution of the two processes, namely the potential overlap of PtO_Red_ and ORR occurs over a wide pH range and therefore, Pt microelectrodes cannot be used in O_2_‐generating reactions such as the OER for local pH determination.^[^ ^14^ ^]^

To overcome this limitation, we thought of an alternative SECM tip as a voltammetric micro/nanosensor probe comprised of Au, which exhibits inferior ORR activity and, thus, well‐separated peaks for the ORR and the reduction of Au—O_*x*_ (Au—O_*x*Red_). The phase‐dependency of Au—O_*x*_ species to pH and potential can be predicted from the Pourbaix diagram and indicates thermodynamically stable species being involved in the Au/Au—O_*x*_ redox process,^[^ ^19^ ^]^ in addition to the known‐super Nernstian correlation. To verify this, CV measurements were performed at a Au tip electrode in 1 m KOH (mainly Au/Au(OH)_3_ process) and 1 m HClO_4_ (mainly Au/Au_2_O_3_ process) (Figure 1b). In both cases, the characteristic oxidation peaks of Au were observed in addition to a sharp reduction peak of Au(OH)_3_ at ≈0 mV versus Ag/AgCl/3 m KCl in 1 m KOH and of Au_2_O_3_ at ≈960 mV vs Ag/AgCl/3 m KCl in 1 m HClO_4_.^[15a]^ In acidic media, the cathodic peak is smaller than the anodic one, which may be due to the dissolution of Au_2_O_3_, considering its instability.^[^ ^20^ ^]^ However, these reduction peaks of Au—O_*x*_ are well distinguishable from the ORR redox wave, which emerges at around 65 mV vs Ag/AgCl/3 m KCl in acid and ≈160 mV vs Ag/AgCl/3 m KCl in alkaline media, making the Au tip a suitable candidate for probing local pH modulations during water oxidation reactions.

**Figure 2:**
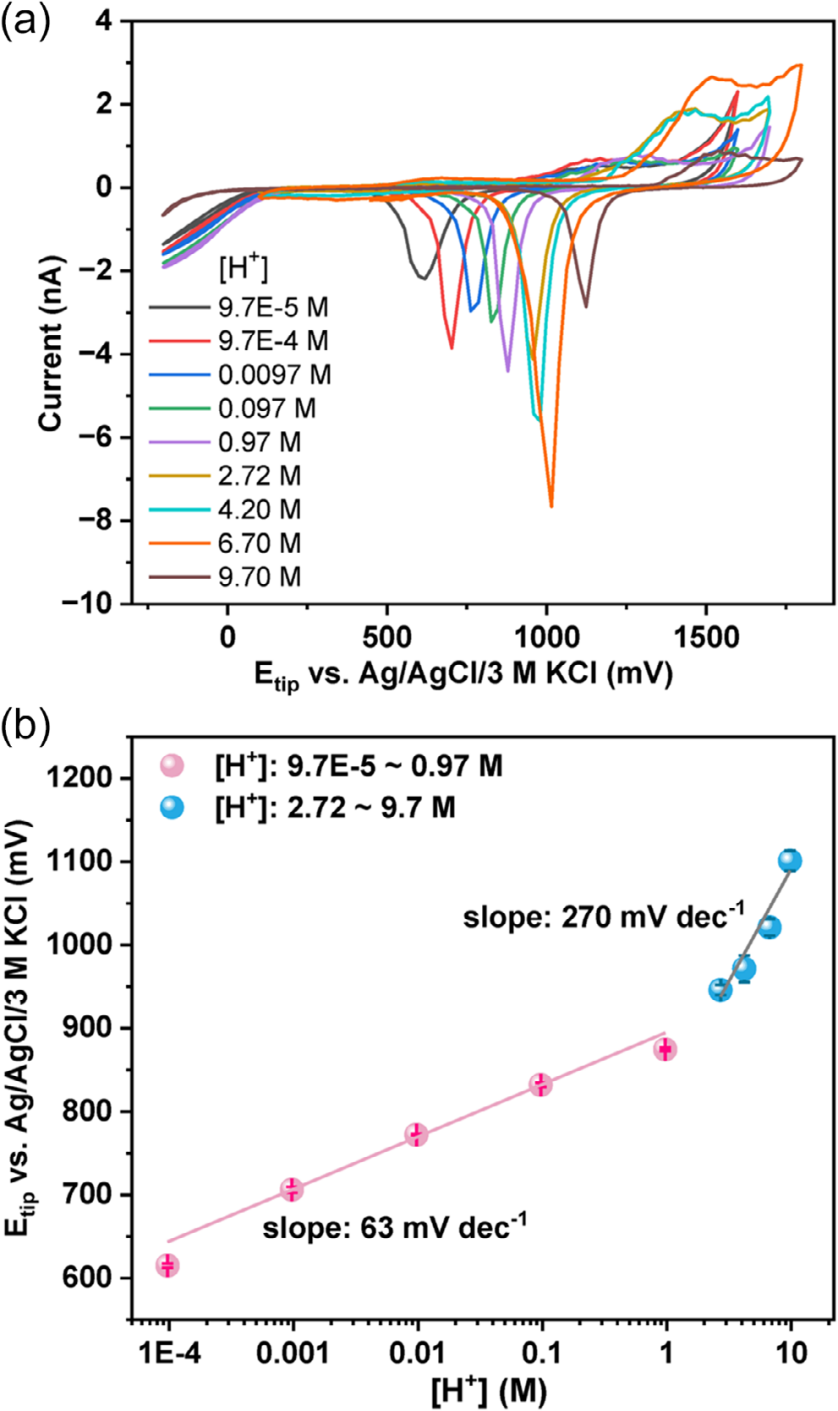
a) pH‐dependent CVs of a bare Au microelectrode (diameter 10 μm) in a series of HClO_4_ solutions with concentrations ranging from 97 μM to 9.7 m. The scan rate is 200 mV s^−1^. b) Calibration curve derived from the peak potential of the Au_2_O_3Red_ as a function of the H^+^ concentration. The peak position of Au_2_O_3Red_ is averaged over three different measurements as indicated by the error bars. The *R* ^2^ of the fitting curves for the proton concentration range of 9.7 E‐5–0.97 m and 2.72–9.7 mare 0.98 and 0.94, respectively.

Prior to the evaluation of the local pH changes at the interface of a catalyst, a calibration curve was established by conducting CVs at the Au tip in a series of differently concentrated HClO_4_ solutions (97 μM to 9.7 m) as shown in **Figure** **2**a. The calibration curve is presented in Figure 2b and demonstrates the pH sensitivity and stability of the Au—O_*x*_ reduction process even in extremely acidic environments. Interestingly, two different slopes are visible within the investigated concentration range, namely 63 mV dec^−1^ for H^+^ concentrations between 97 μM to 0.97 m, and 270 mV dec^−1^ for higher H^+^ concentrations up to 9.7 m. Within this calibration window, no change in the thermodynamically stable Au/Au—O_*x*_ phase occurs and hence the dependency of the Au/Au—O_*x*_ redox potential on the H^+^ and H_2_O activity determines the observed peak potential shift,^[15a]^ as predicted in Equation (3). Here, the tip response was correlated with the H^+^ concentration from the calibration experiment instead of the H^+^ activity in accordance with the pH definition. The significantly increased slope at highly acidic conditions can be explained by a notable increase in the H^+^ activity coefficient,^[^ ^21^ ^]^ where the protons cannot be completely solvated because the available free water molecules become locally limiting. Concordantly, the water activity was determined previously to be less than unity in concentrated HClO_4_ solutions.^[^ ^22^ ^]^

**Figure 3:**
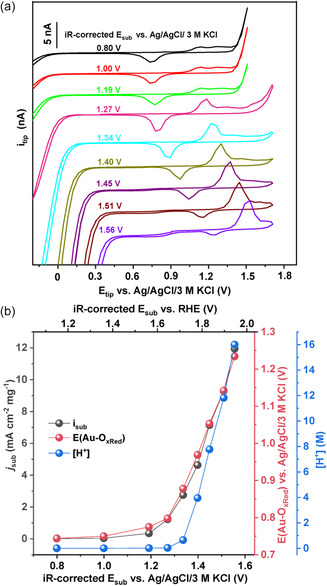
a) CVs recorded at the Au tip in close proximity to the BDD/IrO_2_ catalyst electrode when a series of potential steps were applied to the substrate in 0.005 m HClO_4_ solution. b) Extracted values of substrate current, E(Au—O_*x*Red_), and calculated [H^+^] values as a function of applied potential at the BDD/IrO_2_ anode.

To demonstrate the capability of Au microprobes for local pH sensing in acid conditions, the OER reaction at an IrO_2_ catalyst deposited on a flat boron‐doped diamond (BDD) surface^[^ ^23^ ^]^ was initially studied as a model case representing a catalyst with high OER activity. The SECM tip was positioned close to the surface to guarantee that the SECM tip was probing the diffusion layer in front of the working catalyst surface, as described in the experimental section. The voltammetric responses of the Au tip were recorded while the IrO_2_‐modified BDD surface was polarized at a constant potential (**Figure** **3**a). According to the Nernst equation, a positive potential shift of the Au—O_*x*Red_ peak is expected at the reaction interface with the gradual enrichment of protons, as the substrate is polarized to more positive potentials for the electrochemical water oxidation. The Au—O_*x*Red_ peak shows a slight positive shift from 0.74 to 0.78 V vs Ag/AgCl/3 m KCl when the substrate potential was stepped from 0.8 to 1.2 V versus Ag/AgCl/3 m KCl, indicating a mild pH change.

Such a slight acidification process should be related to the oxidation of the Ir catalyst, which is observed in the CV (Figure S1, Supporting Information). A significant shift of the Au—O_*x*Red_ peak was recorded at a higher substrate potential of more than 1.3 V versus Ag/AgCl/3 m KCl, which indicates that a substantial quantity of protons is generated at the reaction interface, leading to an exceedingly acidic local environment. In parallel, the HER potentials shifted anodically due to the same pH dependency. The cathodic peak decreases in intensity with increasing sample potential while the anodic peak increases, along with a decrease in the peak‐to‐peak separation. Possibly, the oxide formation and its dissolution are favored at the high local O_2_ partial pressure and increased acidification caused by the OER at the sample. The potential shift is quantitatively converted to H^+^ concentrations (Figure 3b) with the E(Au—O_*x*Red_) of the tip changing from 0.76 to 1.23 V versus Ag/AgCl/3 m KCl while the BDD/IrO_2_ substrate was stepped from 1.2 to 1.8 V versus Ag/AgCl/3 m KCl promoting OER. Based on the calibration curve, the local concentration of protons increased from 0.005 to 16 m, while the current density at the substrate increased from 0 to 12 mA cm^−2^ mg^−1^, concordant with the predicted expectations of local acidification due to the OER. The local pH changes in the previously described catalyst system were prominent, however, there are reaction conditions in which the use of a buffering electrolyte may partially compensate for the pH change in the vicinity of the catalyst surface. Although tracking H^+^ activity changes at the catalyst surface in such buffering cases is more of a challenge, it is an important prerequisite as the OER mechanism and kinetics are highly dependent on pH.^[^ ^3^ ^]^ To look into such a scenario, a carbon fiber paper (CFP)‐supported ZnGa_2_O_4_ catalyst (CFP/ZnGa_2_O_4_) was further investigated in a carbonate‐based buffer solution, and the corresponding linear sweep voltammograms can be found in Figure S2, Supporting Information.

In contrast to IrO_2_, ZnGa_2_O_4_ exhibits poor OER activity,^[^ ^24^ ^]^ hence the small amount of H^+^ ions produced will possibly get buffered by the carbonate buffer, depending on the transport phenomena and thermodynamic equilibrium. Besides, porous substrates such as CFP are unsuitable for performing an electrochemical approach (relying on a mediator or a diffusion‐limited reaction taking place at the tip) which is used in conventional SECM. These issues can be circumvented with the employment of non‐electrochemical approach methods such as shear‐force SECM. The latter is a mediator‐free technique that allows the control of tip‐to‐sample distance at a nanometer range using needle‐shaped nanoelectrodes.^[13a]^ The calibration curve of the SECM tip in basic media is shown in Figure S3, Supporting Information. Similar to the first case study on IrO_2_, SECM was employed in which the variations of H_2_O/H^+^ activities were monitored by the modulation of the Au/Au—O_*x*Red_ potential while the catalyst was biased at different potentials to test different OER rates.

**Figure 4:**
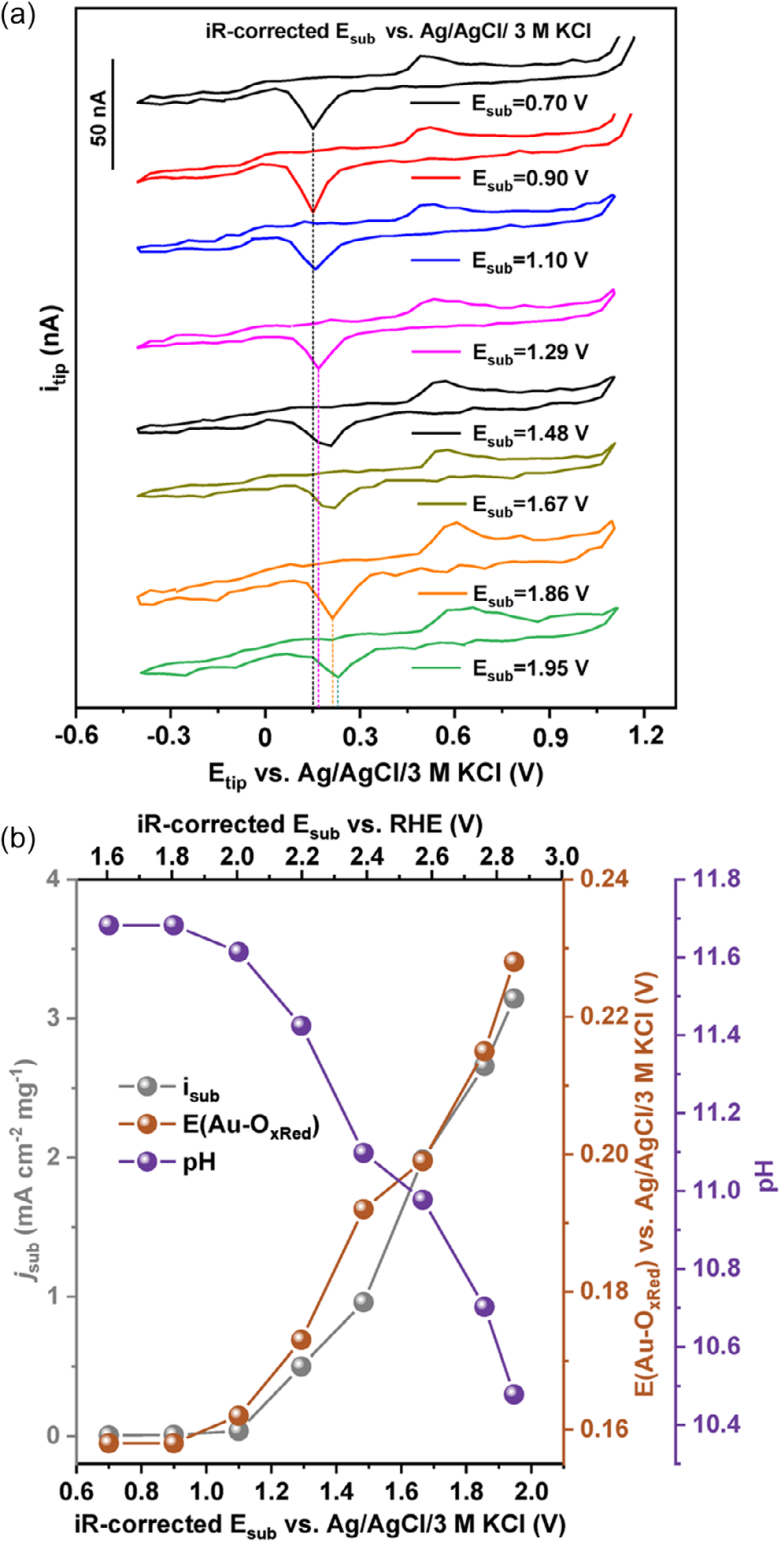
a) CVs were recorded at the Au tip near the CFP/ZnGa_2_O_4_ catalyst when a series of potential steps were applied to the substrate in a carbonate buffer with a bulk pH of 11.7. b) Extracted values of substrate current, E(Au—O_*x*Red_), and calculated pH values as a function of the applied substrate potentials.

**Figure** **4**a presents the CVs recorded at the tip positioned close to the anode. An anodic shift of E(Au/Au—O_*x*Red_) was observed from 0.16 to 0.23 V when the substrate was polarized from 1.1 to 1.95 V vs Ag/AgCl/3 m KCl (Figure 4b). Interestingly, the water oxidation current density at the substrate increased from 0 to 3.1 mA cm^−2^ mg^−1^ while the local pH changed from 11.7 to 10.5 in the highly buffered 2 m bicarbonate‐based electrolyte. The small variation in pH here showed that the pH shift in the alkaline buffer solution is partially compensated by the HCO_3_^−^/CO_3_^2−^ equilibrium, revealing the high sensitivity of the bare Au to probe pH changes.

## 3 Conclusion

We elucidated the aptness of bare gold micro/nanoelectrodes as SECM tips for local pH sensing during the OER. The capability of this approach is demonstrated by investigating local pH changes above two OER catalysts in different electrolyte environments. Initially in the BDD/IrO_2_ investigation, we observed an abrupt change of proton concentration in the vicinity of the catalyst, from 0 to 16 m while the substrate was swept to more positive OER potentials. The large shift of SECM tip response depicted the local enrichment with H^+^ ions due to the highly active OER catalyst and the absence of buffering species in the electrolyte. This result demonstrates the stability of the bare Au electrode at highly acidic conditions. In the buffered alkaline condition, a decrease in the local pH from 11.7 to 10.5 was determined close to a CFP/ZnGa_2_O_4_ catalyst. The small pH variation could be an indicator of the low electrochemical activity of the catalyst towards the OER and/or the high buffering capacity of the electrolyte which mitigated the pH drop. In these two studied cases, we could see the wide range of applicability of the Au probes in cases where the local changes in acidity were prominent, as well as in alkaline buffering solutions. Local pH tracking during water splitting in proximity to the catalyst interface can open the door for a deeper understanding of these processes and the catalyst itself, ultimately speeding up catalyst development. The proposed method is suitable for mapping local sample heterogeneities which would require a mapping protocol including all measurement steps that were introduced in this work.

## 4 Experimental Section

4.1

4.1.1

##### Preparation of IrO_2_ and ZnG_2_O_4_ Anodes

Commercial IrO_2_ (99.9%) was purchased from Sigma‐Aldrich. 5 mg IrO_2_ was dispersed in a 1 mL solution consisting of distilled water and isopropanol (V/V = 1/1). The mixture was treated with tip sonication for 40 min, and then 5 μL of a 5 wt% Nafion solution was added. After being treated with bath sonication for 40 min, 10 μL of the obtained ink was drop cast onto a boron‐doped diamond substrate^[^ ^23^ ^]^ with an exposed surface area of 0.196 cm^2^ and dried at room temperature (RT).

The ZnGa_2_O_4_ catalyst was prepared with minor modifications according to a previously reported hydrothermal method.^[^ ^25^ ^]^ A 10 mL mixture solution containing 50 mM Ga^3+^ and 25 mM Zn^2+^ was prepared using gallium nitrate hydrate and zinc acetate. Then, 5 mL of ethylenediamine was added and the solution was stirred at RT for 20 min. After that, the mixture was transferred into an autoclave (25 mL) and heated at 200 °C for 16 h. After passively cooling down to RT, the white powder was collected by centrifugation, and washed with deionized water and with ethanol several times. Then the obtained powder was dried at 60 °C overnight. 50 mg ZnGa_2_O_4_ catalyst and 10 mg polytetrafluoroethylene were dispersed in 10 mL ethanol, followed by bath sonication for 20 min to form a highly dispersed mixture. Then the ZnGa_2_O_4_ catalyst mixture was deposited onto CFP (Toray H‐060) by spray coating. The CFP was cleaned with ethanol before use, and the cleaned CFP was placed on a hot plate (100 °C) during spray coating. The loading of ZnGa_2_O_4_ was about 10 mg cm^−2^.

##### Calibration Curve of E(Au—O_xRed_) at Different pH Conditions

A calibration curve for E(Au—O_*x*Red_) as a function of proton concentration was established. This was accomplished by recording the potentiodynamic response of the Au tip in a series of perchloric acid (HClO_4_) solutions with varying concentrations (97, 970 μM, 9.7, 97 mM, 0.97, 2.72, 4.2, 6.7, and 9.7 m). These solutions were prepared by diluting a stock solution with a nominal concentration of 11.7 m HClO_4_ (99.9% trace metal basis, Sigma Aldrich). The exact concentrations of HClO_4_ solutions were subsequently determined through acid‐base titration. Firstly, a 36.1 mM oxalic aqueous solution was prepared by dissolving 455.24 mg of oxalic acid dihydrate in a 100 mL volumetric flask. A nominal 0.1 m potassium hydroxide (KOH) solution was also prepared. Three aliquots of 10 mL oxalic acid solution were prepared, and five drops of phenolphthalein were added to each aliquot as an indicator. The glass burette was thoroughly rinsed and filled with the nominal 0.1 m KOH solution up to its upper mark. Subsequently, three individual titration experiments were conducted. In each experiment, the KOH titrant was incrementally added drop by drop to a predetermined aliquot of the oxalic acid solution until a consistent pinkish coloration persisted for more than 10 s. The volume of titrant consumed was recorded for each trial, and an average value was calculated based on these three separate samples. The actual concentration of KOH was determined to be 0.1014 m.

Secondly, the exact concentration of HClO_4_ with a nominal concentration of 0.1 m was determined following the steps: three aliquots of 10 mL HClO_4_ solution were prepared, with five drops of phenolphthalein added as an indicator. Each titration was conducted by incrementally adding the KOH titrant to the HClO_4_ solution until a consistent pinkish coloration persisted for more than 10 s. Finally, the accurate concentration of HClO_4_ was calculated to be 0.097 m.

##### SECM Measurements

A home‐made SECM set‐up was positioned inside a Faraday cage, the walls of which were isolated with vacuumed polystyrene panels (Vaku Isotherm) to prevent deviations in temperature, while the vibrational noise was prevented by putting the Faraday cage on an actively damped table (Newport RS 2000).

The SECM measurement of the IrO_2_ catalyst was done by placing the 10 μm Au ultramicroelectrode (UME) in proximity to the catalyst surface by performing a negative feedback SECM approach curve using the diffusion‐limited reduction process of O_2_ dissolved in the electrolyte. The approach curves were recorded in O_2_‐saturated 0.005 m HClO_4_ solution, the Au tip was polarized at −450 mV vs Ag/AgCl/3 m KCl to reduce O_2_. The tip current decreases as the tip gets closer to the surface since the diffusional access of O_2_ to the SECM will be more and more hindered by the substrate surface. Once the current reaches a minimum, the approach is stopped and that will be the position of the UME during the following experiment. The working distance for the local pH monitoring experiment was around 10 μm.

The experiments with the ZnGa_2_O_4_ catalyst were done by shear force approach with a nanoelectrode with a diameter of 650 nm (fabrication procedure reported previously^[^ ^26^ ^]^), to get closer to a nm range to the sample. Two piezos (Piezomechanik Pickelmann) were mounted at the electrode, one closer to the tip (detection piezo) and the other one ≈2 cm away (excitation piezo) at a 45^o^ angle, both connected to a lock‐in amplifier (Ametek 7280). While the excitation piezo applies AC voltages to the tip to induce its oscillation, the detection piezo measures the phase and magnitude of the oscillation. In the presented experiments the oscillation magnitude served as the feedback parameter for the distance control. A detailed experimental description of shear‐force SECM is given elsewhere.[^13^, ^27^]

Once the Au tip is approached to the sample, CVs are recorded at the tip in all experiments while the substrate is biased at different potentials, starting from 500 mV up to 2000 mV vs Ag/AgCl /3 m KCl. More specifically, initially a potential at which no Faraday current is observed (500 mV vs Ag/AgCl/3 m KCl) was applied to the sample until the background current stabilized. Thereafter, a predefined OER potential was applied to the sample while simultaneously recording CVs to the micro‐or nanoelectrode until they overlap. Finally, the resting potential was applied once again to ensure that the CV matched the one before the applied OER potential. This ensures that the bulk pH is reestablished between subsequent measurements.

All experiments were performed with a Pt mesh as a counter electrode (CE) and a homemade Ag/AgCl/3 m KCl as a reference electrode (RE).

##### Materials Characterization

Scanning electron microscopy images were recorded using a Quanta 3D ESEM (FEI) at 20 kV acceleration voltage in the high vacuum mode. Potentials set against Ag/AgCl/3 m KCl were converted to the RHE scale according to *E*_RHE_ = *E*_Ag/AgCl_ + 0.21 + 0.059 × pH, and the bulk pH values of electrolytes were tested by a pH meter (FE28, Mettler Toledo).

## Conflict of Interest

The authors declare no conflict of interest.

## Supporting information

Supplementary Material

## Data Availability

The data that support the findings of this study are available from the corresponding author upon reasonable request.

## References

[1] J. P. Lange , Angew. Chem. Int. Ed. Engl. 2015, 54, 13186. 26457585 10.1002/anie.201503595

[2] a) A. Raveendran , M. Chandran , R. Dhanusuraman , RSC Adv. 2023, 13, 3843; 36756592 10.1039/d2ra07642j PMC9890951

[3] J. C. Fornaciari , L.‐C. Weng , S. M. Alia , C. Zhan , T. A. Pham , A. T. Bell , T. Ogitsu , N. Danilovic , A. Z. Weber , Electrochim. Acta 2022, 405, 139810.

[4] W. Chen , M.‐K. Zhang , B.‐Y. Liu , J. Cai , Y.‐X. Chen , Curr. Opin. Electrochem. 2022, 34, 101003.

[5] a) Y. Yokoyama , K. Miyazaki , Y. Miyahara , T. Fukutsuka , T. Abe , ChemElectroChem 2019, 6, 4750;

[6] C. S. Santos , A. S. Lima , D. Battistel , S. Daniele , M. Bertotti , Electroanalysis 2016, 28, 1441.

[7] a) M. C. O. Monteiro , L. Jacobse , T. Touzalin , M. T. M. Koper , Anal. Chem. 2020, 92, 2237; 31874560 10.1021/acs.analchem.9b04952 PMC6977089

[8] M. C. O. Monteiro , M. T. M. Koper , Curr. Opin. Electrochem. 2021, 25, 100649.

[9] G. Seiffarth , M. Steimecke , T. Walther , M. Kühhirt , S. Rümmler , M. Bron , Electroanalysis 2016, 28, 2335.

[10] N. Limani , A. Boudet , N. Blanchard , B. Jousselme , R. Cornut , Chem. Sci. 2020, 12, 71. 34163583 10.1039/d0sc04319b PMC8178752

[11] M. Michalak , M. Kurel , J. Jedraszko , D. Toczydlowska , G. Wittstock , M. Opallo , W. Nogala , Anal. Chem. 2015, 87, 11641. 26516786 10.1021/acs.analchem.5b03482

[12] M. C. O. Monteiro , A. Mirabal , L. Jacobse , K. Doblhoff‐Dier , S. C. Barton , M. T. M. Koper , JACS Au 2021, 1, 1915. 34849509 10.1021/jacsau.1c00289 PMC8611793

[13] a) S. Dieckhofer , D. Ohl , J. R. C. Junqueira , T. Quast , T. Turek , W. Schuhmann , Chem. Eur. J. 2021, 27, 5906; 33527522 10.1002/chem.202100387 PMC8048634

[14] A. Botz , J. Clausmeyer , D. Ohl , T. Tarnev , D. Franzen , T. Turek , W. Schuhmann , Angew. Chem. Int. Ed. Engl. 2018, 57, 12285. 30073732 10.1002/anie.201807798

[15] a) S. Yang , D. G. H. Hetterscheid , ACS Catal. 2020, 10, 12582; 10.1021/acscatal.0c00531 PMC7137537 32280560

[16] a) Y. Shao , M. V. Mirkin , Anal. Chem. 1997, 69, 1627;

[17] L. W. Liao , M. F. Li , J. Kang , D. Chen , Y.‐X. Chen , S. Ye , J. Electroanal. Chem. 2013, 688, 207.

[18] O. Rodríguez , G. Denuault , ChemElectroChem 2021, 8, 3525.

[19] M. Pourbaix , Electrochemical Equilibria In Aqueous Solutions, National Association of Corrosion Engineers, New York 1974.

[20] a) S. Cherevko , A. R. Zeradjanin , G. P. Keeley , K. J. J. Mayrhofer , J. Electrochem. Soc. 2014, 161, H822;

[21] C. G. McCarty , E. Vitz , JCE DigiDemos 2006, 83, 752.

[22] H. Wai , K. Yates , Can. J. Chem. 1969, 47, 2326.

[23] N. Limani , A. Boudet , E. Scorsone , V. Derycke , B. Jousselme , R. Cornut , Electrochem. Commun. 2023, 153, 107538.

[24] L. Li , Z. Hu , Y. Kang , S. Cao , L. Xu , L. Yu , L. Zhang , J. C. Yu , Nat. Commun. 2023, 14, 1890. 37019917 10.1038/s41467-023-37007-9 PMC10076521

[25] Q. Liu , D. Wu , Y. Zhou , H. Su , R. Wang , C. Zhang , S. Yan , M. Xiao , Z. Zou , ACS Appl. Mater. Interfaces 2014, 6, 2356. 24475972 10.1021/am404572g

[26] M. C. O. Monteiro , S. Dieckhofer , T. Bobrowski , T. Quast , D. Pavesi , M. T. M. Koper , W. Schuhmann , Chem. Sci. 2021, 12, 15682. 35003599 10.1039/d1sc05519d PMC8654039

[27] B. Ballesteros Katemann , A. Schulte , W. Schuhmann , Chem. Eur. J. 2003, 9, 2025. 12740850 10.1002/chem.200204267

