# Supplementary material for: Au Micro‐ and Nanoelectrodes as Local Voltammetric pH Sensors During Oxygen Evolution at Electrocatalyst‐Modified Electrodes
Source: Small Sci. 2024 Feb 12;4(4):2300283. doi: 10.1002/smsc.202300283 (PMC11935127; doi:10.1002/smsc.202300283)
Supplement: Supplementary file 1 — Supplementary Material [file SMSC-4-2300283-s001.pdf]

Supporting information

Au Micro- and Nanoelectrodes as Local Voltammetric pH Sensors during Oxygen Evolution at Electrocatalyst-modified Electrodes

L. Li,[+] N. Limani,[+] R. Antony, S. Dieckhöfer, C. Santana Santos, W. Schuhmann*

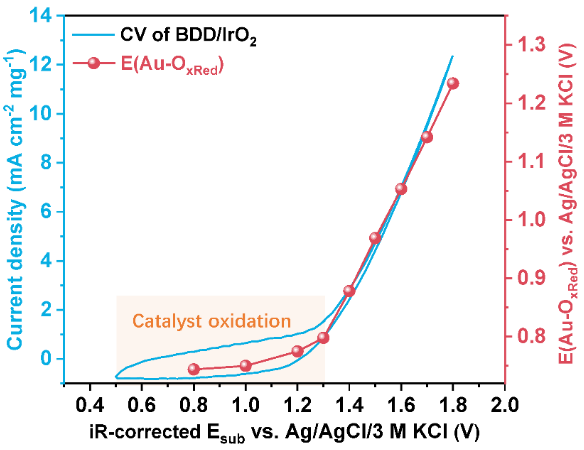

Figure S1. Cyclic voltammetry of BDD/IrO_2 in 0.005 M $HClO_4$ solution and the peak potential of Au_2O_{3Red} as a function of the substrate potential.

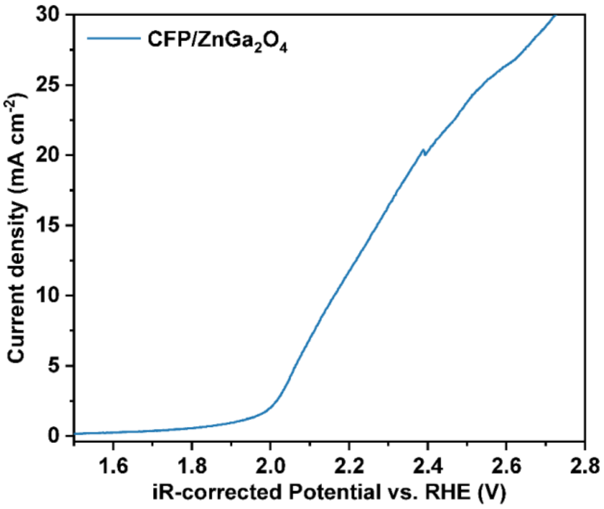

Figure S2. Linear sweep voltammogram of a $CFP/ZnGa_2O_4$ anode in carbonate-based buffer solution at a pH of 11.7. The scan rate is 20 mV s^{-1}.

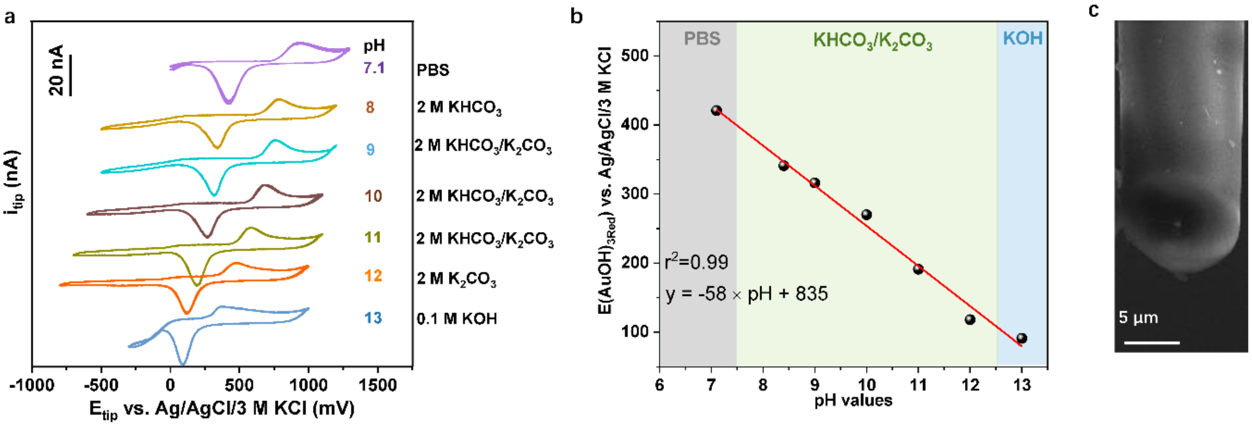

Figure S3. (a) Au tip CVs recorded at different pH values. (b) The linear fits of the relationship between E(Au(OH)$_{3Red}$) and the solution pH values. (c) SEM image of the Au nanoelectrode used for local pH measurements. The CVs of the Au tip were recorded in solutions in a pH range from 7.1 to 13 and the calibration curve extracted from the CVs is shown in (b).

In near-neutral and alkaline media, the oxidation of Au can be described as

$$Au + 3H_2O \rightarrow Au(OH)_3 + 3H^+ + 3e^-.$$

Therefore, as shown in the equation below, the Au/Au(OH)$_3$ presents a pH dependence.

$$E_{Au(OH)3/Au} = E_0 - \frac{RT}{3F}\ln\frac{a(Au)\cdot a(H_2O)^3}{a(Au\ (OH)_3)\cdot a(H^+)^3}$$

The E(Au(OH)$_{3Red}$) shows a distinct anodic shift with the increasing activity of protons and decreasing activity of water and the fit exhibits a super Nernstian pH dependence in the tested pH range.
